# Editorial: Pathophysiology and treatment of fusiform intracranial aneurysms

**DOI:** 10.3389/fneur.2022.893712

**Published:** 2022-08-22

**Authors:** Behnam Rezai Jahromi, Felix Göhre, Riikka Tulamo, Hugo Andrade-Barazarte

**Affiliations:** ^1^Helsinki University Central Hospital, Helsinki, Finland; ^2^Department of Neurosurgery, Helsinki University Central Hospital, Helsinki, Finland; ^3^Neurosurgery Research Group, Biomedicum, Helsinki University Hospital, University of Helsinki, Helsinki, Finland; ^4^Neurochirurgie BG Klinikum Bergmannstrost, Halle, Germany; ^5^Department of Vascular Surgery, Helsinki University Hospital, University of Helsinki, Helsinki, Finland; ^6^Division of Neurosurgery, Department of Surgery, University of Toronto, Toronto, ON, Canada

**Keywords:** aneurysm, fusiform, dolichoectasia, basilar aneurysms, Vertebrobasilar Aneurysm

Fusiform intracranial aneurysms (fIA), unlike their sister pathology saccular intracranial aneurysms (sIA), lack a defined neck to be closed from circulation. They are predominantly seen more in male patients than female, especially in those patients who suffer from other cardiovascular disorders. Genetic pathways of fIAs and sIAs are somehow overlapping, but unlike sIAs, fIAs are more frequent within the posterior circulation (1). The multiple terms used to describe this type of non-saccular aneurysms (fusiform, translational, or dolichoectatic aneurysms) reflect their poorly known and understood developmental history, as well as their prognosis and pathophysiology. Treatment of fiAs has been challenging despite the development of endovascular procedures and state-of-the-art microsurgical revascularization techniques. Sudden flow changes and the tortuous structure of fIAs has made their current treatment ineffective, with high morbidity and mortality. This poor outcome is related to their location within the posterior circulation and basilar artery (BA) since these arteries provide important feeders to the brainstem.

Moreover, excluding fIA from the circulation has been a true challenge, only a few biopsies have been available for our community to research on. For example, the elegant technique reported by Narsinh et al. in “Endovascular Biopsy of Vertebrobasilar Aneurysm in Patient with Polyarteritis Nodosa”, demonstrated and opens a new biopsy possibility for data and histological sample collection that could be used to understand not just fIAs, but other major intracranial pathologies.

The true etiology of fIAs remains still unknown, it is believed that the basilar artery diameter has an impact on patients' symptoms and outcome, a diameter of 4.5 mm increases the risk of stroke, whereas a diameter of more than 10 mm increases the risk of rupture.

Management of symptomatic fIAs includes endovascular techniques and microsurgery. The aim of endovascular stenting is to deploy a perfectly sized stent that adheres to the arterial wall causing hemodynamic stability of the pathology. Additionally, it also attracts endothelial cells from healthy segments of the arterial wall without occluding small perforators. Furthermore, this is not always a straightforward procedure, it requires considering multiple variables before implanting the stent and achieving a benign hemodynamical environment.

Hemodynamics play a special role in intracranial aneurysm formation, it is thought that it has an impact on aneurysm wall inflammation and it can have a degenerative role and transformation of an unruptured aneurysm into a ruptured one. Saalfeld et al. studied the hemodynamic changes in virtual reality in order to identify the best option between the different endovascular modalities for inserting stents that provided the best hemodynamic option for subsequent arterial wall healing. This method is important since it allows us to identify and assess other treatment alternatives such as the revascularization procedures of Dolichoectatic aneurysms since many of these non-saccular aneurysms are pathological in all arterial segments with a complex, tortuous, and uneven wall structure.

The aim of microneurosurgical treatment of fIAs is usually to preserve perforators toward the brainstem and induce hemodynamic stabilization within the whole aneurysmatic segment, however, this is rarely achieved through traditional clipping techniques. Izumo et al. demonstrated an elegant case where the rupture point of dolichoectasia was separately clipped while the rest of the pathological segment was left untreated. Moreover, the posterior circulation predominance of these aneurysms makes their treatment challenging even through endovascular methods. Therefore, treatment decisions should be discussed in multidisciplinary clinics and based on patient and aneurysm characteristics. Also, Tsunoda and Inoue presented their microsurgical treatment strategy for fusiform vertebral artery aneurysms focusing on hemodynamic integrity and perforator preservation with fine microsurgical techniques. In particular, the importance of bypass techniques was shown.

Additionally, Guo et al. showed the difficulty in completely defining non-saccular aneurysms as compared to saccular ones, since fIAs usually have heterogenous arteriopathy.

Despite the challenge that non-saccular aneurysms bring to neurosurgeons, there have been developments for the better. To continue this pathway there is a need for more collaboration internationally to find the best and most innovative treatment options in the future.

## Author contributions

All authors have contributed to writing and design of editorial and topic.

## Conflict of interest

The authors declare that the research was conducted in the absence of any commercial or financial relationships that could be construed as a potential conflict of interest.

## Publisher's note

All claims expressed in this article are solely those of the authors and do not necessarily represent those of their affiliated organizations, or those of the publisher, the editors and the reviewers. Any product that may be evaluated in this article, or claim that may be made by its manufacturer, is not guaranteed or endorsed by the publisher.

## References

[1] Rezai Jahromi B Valori M Tulamo R Jauhiainen S Ilmonen H Kantonen J. Cancer-type somatic mutations in saccular cerebral aneurysms. SSRN Electron J. (2021). 10.2139/ssrn.3961687 PMC12322075 39668185

